# Genetics and Epigenetics of One-Carbon Metabolism Pathway in Autism Spectrum Disorder: A Sex-Specific Brain Epigenome?

**DOI:** 10.3390/genes12050782

**Published:** 2021-05-20

**Authors:** Veronica Tisato, Juliana A. Silva, Giovanna Longo, Ines Gallo, Ajay V. Singh, Daniela Milani, Donato Gemmati

**Affiliations:** 1 Department of Translational Medicine and LTTA Centre, University of Ferrara, 44121 Ferrara, Italy; daniela.milani@unife.it; 2 University Center for Studies on Gender Medicine, University of Ferrara, 44121 Ferrara, Italy; 3 Department of Translational Medicine, University of Ferrara, 44121 Ferrara, Italy; slvjln@unife.it (J.A.S.); giovanna.longo@unife.it (G.L.); ines.gallo@unife.it (I.G.); 4 Physical Intelligence Department, Max Planck Institute for Intelligent Systems, 70569 Stuttgart, Germany; Ajay-Vikram.Singh@bfr.bund.de; 5 Department of Chemical and Product Safety German Federal Institute (BfR), Max-Dohrnstr 8-10, 10589 Berlin, Germany; 6 Centre of Hemostasis & Thrombosis, University of Ferrara, 44121 Ferrara, Italy

**Keywords:** brain-epigenome, one-carbon metabolism genes, folate, SNPs, gene variants, epigenetics, autism spectrum disorder (ASD), sex-gap, gender-gap

## Abstract

Autism spectrum disorder (ASD) is a complex neurodevelopmental condition affecting behavior and communication, presenting with extremely different clinical phenotypes and features. ASD etiology is composite and multifaceted with several causes and risk factors responsible for different individual disease pathophysiological processes and clinical phenotypes. From a genetic and epigenetic side, several candidate genes have been reported as potentially linked to ASD, which can be detected in about 10–25% of patients. Folate gene polymorphisms have been previously associated with other psychiatric and neurodegenerative diseases, mainly focused on gene variants in the *DHFR* gene (5q14.1; rs70991108, 19bp ins/del), *MTHFR* gene (1p36.22; rs1801133, C677T and rs1801131, A1298C), and *CBS* gene (21q22.3; rs876657421, 844ins68). Of note, their roles have been scarcely investigated from a sex/gender viewpoint, though ASD is characterized by a strong sex gap in onset-risk and progression. The aim of the present review is to point out the molecular mechanisms related to intracellular folate recycling affecting in turn remethylation and transsulfuration pathways having potential effects on ASD. Brain epigenome during fetal life necessarily reflects the sex-dependent different imprint of the genome-environment interactions which effects are difficult to decrypt. We here will focus on the *DHFR*, *MTHFR* and *CBS* gene-triad by dissecting their roles in a sex-oriented view, primarily to bring new perspectives in ASD epigenetics.

## 1. Introduction

In 1943, Kanner was the first to systematically define autism as an innate inability to create normal, biologically determined, and emotional contact with others [1]. Currently, autistic disorder, along with pervasive developmental disorder not otherwise specified (PDD–NOS), and Asperger syndrome represent the complex set of human neurodevelopmental diseases collectively known as autism spectrum disorder (ASD) [2]. Although potentially diagnosed at any age, autism has an early age of onset with more than 1% of affected children characterized by a wide range of severity and continuous distribution of ASD traits in the general population [3]. ASD patients typically experience difficulty with social communication and interaction, restricted interests, and repetitive behaviors [4]. Indeed, despite the available technological advances and the innovative experimental approach of study, there is still not a definite and direct causal relationship or pattern to completely understand the pathogenesis of this complex disorder [5]. 

On the other hand, there is evidence for specific features in common with other diseases such as immune dysregulation and inflammation characterized by higher pro-inflammatory cytokines (mirror of neuroinflammation), oxidative stress inception and mitochondrial dysfunction as well as dysfunctions of other organs than the brain (e.g., gastrointestinal disorders) [6,7,8]. Although it is not clear whether to consider them as disease-causative events, or mere consequence of other etiological processes, they are under investigation as potential targets for ASD treatment [8,9,10].

ASD has a strong heritable component making it essentially a genome-based human disease [11]. It has been reported indeed that monozygotic twins have around a 90% chance of sharing the disease, while dizygotic twins have only a 5% to 10% risk of comorbidity [12]. Besides that, Fragile X Syndrome (FXS) is reported as the most common X-linked monogenic cause of Intellectual Disability (ID) or ASD, and rare or common gene variants, as well as particular gene deletions in the mother’s genome, might contribute to ASD development [13]. Together with recognized gene-linked syndromes, several ASD patients show chromosomal rearrangements [14], with a crucial time point for damage-onset identified during embryogenesis at the stage of neural tube closure [15]. Of note, this event has been hypothesized to have sex-related differences due to the role of specific genes (i.e., *SOX9*) in affecting male phenotype development and skeletal growth [16,17]. So far, incomplete penetrance has been observed and no causative specific gene has been definitely demonstrated to be the primary ASD contributor [13].

GWAS are now bridging the gap in knowledge existing between ASD and other neurodevelopmental/neuropsychiatric disorders, with the latter being more extensively investigated. Accordingly, the existence of 12 independent loci significantly associated with attention-deficit/hyperactivity disorder (ADHD), a neurodevelopmental psychiatric disorder overlapping ASD, has been reported also highlighting the key role of GWAS in discovering common gene variants [18,19]. Moreover, additional common risk gene variants have been identified as significantly associated with ASD, mainly related to neuronal function and corticogenesis, as well as genetic correlations with other complex disorders and traits in line with a common etiology of the different pathology [20]. Of note, different ASD clinical phenotypes are characterized by complex polygenic architectures and organization [20]. Finally, in the recently published largest ASD exome sequencing study, 102 risk genes have been associated with brain development and regulation of gene expression and neuronal communication [19].

In terms of ASD risk factors definition, there is unanimous agreement on the multifactorial framework of the etiology, suggesting that in most cases autism results from the interaction of multiple genetic and environmental factors, as often demonstrated for other complex diseases [21,22,23,24,25,26,27]. In this line, the role and interplay between genetics and environment with effects on epigenetics and epigenomics have become the subject of intensified researches [5,28,29]. Hypotheses have been attempted to explain the environmental components of ASD, including aspects related to diet and to nutritional epigenetics [30], economic status, vaccination, general health, environmental pollutants, gut, oral and vaginal microbiomes, the latter indicating potential in utero etiopathology of ASD during pregnancy [5,31,32,33]. Overall, despite the several hypotheses, the precise role of genetic and environmental factors in determining the individual risk and disease phenotype still needs to be fully clarified.

Among those genes associated with modification of disease susceptibility, the ones belonging to the folate homeostasis and methionine-homocysteine recycling are gaining interest in the context of different neuropsychiatric/neurobehavioral disorders [34,35]. In the past decades, several studies indicated that low folate levels and vitamin B12 together with high homocysteine (Hcy) levels were associated with neurodevelopmental disease, especially cognitive decline in psychogeriatric and psychiatric patients [36]. This condition may be exacerbated by the presence of specific gene polymorphisms of the folate pathway that drive folate isoforms cycling and balance [37]. In this line, a personalized folic acid supplementation during pregnancy based on the genetic assessment of pregnant women should be considered [37].

Overall, the gene-triad belonging to the remethylation and transsulfuration pathways are dihydrofolate reductase (*DHFR*), methylenetetrahydrofolate reductase (*MTHFR*), and cystathionine-β synthase (*CBS*) are potential candidates as modifier genes in ASD susceptibility.

## 2. One-Carbon Metabolism Pathway

One-carbon metabolism is crucial in epigenetic regulation during embryo development and it is an integrated complex system composed of three main pathways: the folate cycle, the methionine cycle, and the transsulfuration pathway [38]. Folate belongs to the B-vitamins family (B9); it can be obtained from nutritional sources (e.g., leafy greens, beans, vegetables, seeds/legumes) or by supplementation in the form of folic acid. Folate is considered a key factor during neurodevelopment, its deficit associated with neural tube defects (NTD) have led in the recent past to extensive fortification protocols in several countries [39]. Dietary folate is absorbed at the intestinal level by the proton-coupled folate transporter (PCFT) and the reduced folate carrier (RFC), shuttled to the liver via hepatic portal vein followed by hepatocytes uptake. When secreted in blood circulation, folate can reach several tissues and undergo cellular uptake by means of three folate receptors (FR) the GPI-anchored cell membrane FRα and FRβ, and the secreted form FRγ [40]. The receptors are differentially expressed in the different tissues, although FRα plays the main role in cellular uptake [40]. Once in the cells, reduced folate isoforms act as cofactors in the one-carbon units metabolism [41,42]. Importantly, as they serve as one-carbon carriers for methyl group transfer to cytosine residues of CpGs promoter regions in genomic DNA, folate has key roles in DNA synthesis/repair, in purines/pyrimidines synthesis, aminoacid synthesis as well as in DNA/histone methylation, the latter being one of the main epigenetic processes able to change/affect gene expression in both healthy and disease conditions [43,44,45]. Overall, folate is crucial during neurodevelopment and it represents an efficient mediator of the crosstalk between genetics and epigenetics [40,46]. Deficiencies or unbalancing of the mutual levels of the different intracellular folate isoforms may negatively act during fetal growth and promote pediatric cancers, leukemia, neurodevelopmental disorders [47,48,49,50,51].

**Figure 1:**
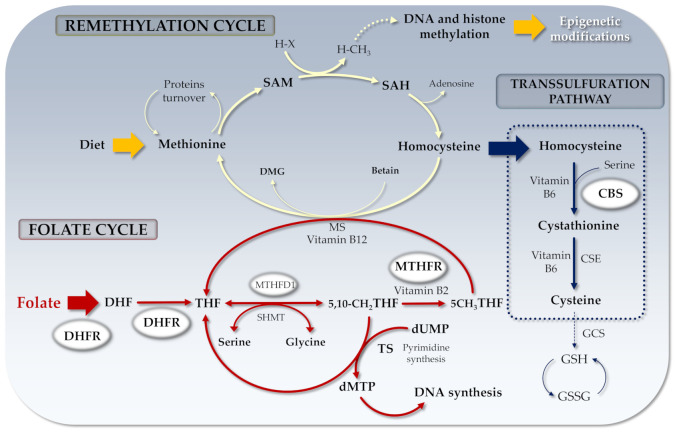
Folate cycle and related genes. DHF: Dihydrofolate; THF: Tetrahydrofolate; SAM: S-adenosyl methionine; SAH: S-adenosyl Homocysteine; MTHFR: Methylenetetrahydrofolate reductase; RCF: Reduced folate carrier; DHFR: Dihydrofolate reductase; dUMP: Uridine monophosphate; dTMP: Thymidine monophosphate; DMG: Dimethylglycine; MS: Methionine synthase; CBS: Cystathionine-β-synthase; H-X: Methyl acceptor; H-CH_3_: Methylated acceptor; TS: Thymidylate synthase; MTHFD1: Methylenetetrahydrofolate dehydrogenase-1 (NADP+ dependent); CSE: Cystathionine γ-lyase; GCS: γ-glutamylcysteine synthetase; GSH: Glutathione; GSSG: Glutathione disulfide.

Folate biochemistry has been deeply investigated in the past [43]. Briefly, once in the cells, folate is converted into its tetrahydrofolate (THF) active form by a two-step process dependent on NADPH and DHFR. As shown in Figure 1, 5,10-methylene-THF can be converted to 5-methyl-THF by the *MTHFR* enzyme and recycled back to THF by methionine synthase (MS). 5-methyl-THF may transfer its methyl group to cobalamin (B12) and the resulting methyl-cobalamin can act as a methyl-donor for Hcy to generate MS-mediated methionine. Finally, the irreversible degradation of Hcy takes place by the transsulfuration pathway via sulfur transfer from Hcy to cysteine by *CBS* enzyme, considered as the only way for cysteine synthesis [52] a potent component of antioxidant glutathione [53].

Males typically show higher Hcy circulating levels compared to females (about 10–15% higher), though the sex gap is normally reduced by women menopause transition. Folate deficiency is associated with hyperhomocysteinemia, a recognized risk factor for several pathological conditions, with a strong inverse relation driven by the number of variant alleles in the *MTHFR* gene [43,49]. In a recent study performed in Israel where no national folic acid fortification programs exist, males, as expected, showed the lowest folate levels compared to females [54]. The consequence of low folate on Hcy levels was that males had higher Hcy than females also in presence of normal B12 levels and independently from age and estrogen effect [54].

Potential explanations may be due to sex-related differences in the prevalence and effects of polymorphic gene variants [55]. Accordingly, anomalous transsulfuration pathway and dysregulated folate or Hcy metabolism may lead to aberrant redox homeostasis and neurodegeneration, and in turn, increased ASD susceptibility [56,57,58]. Several studies have indeed reported a correlation between high Hcy and low folate, vitamin B6, and B12 levels with ASD and severity [58,59]. Of note, gastrointestinal disorders experienced by ASD children may be also due to insufficient intake of B-family vitamins responsible for increased circulating Hcy [60]. As a proof of concept of the proposed link, folate supplementation would result in improved clinical symptoms in ASD patients [61], in reducing Hcy levels, and in glutathione metabolism optimization [62,63].

Changes in levels of metabolites belonging to the methionine and folate cycle can be considered epigenetic predictive biomarkers for ASD as well as potential therapeutic targets though correlations with ASD etiology/severity still need to be defined [64,65]. Overall, in a future perspective, mother-child genomes and epigenetics interactions might become new targets for innovative therapeutic interventions.

### 2.1. MTHFR Gene and Functions

*MTHFR* gene is located on chromosome 1 (1p36.22), it comprises 12 exons and encodes for a protein product of 697aa [66]. Within the folate cycle, the *MTHFR* enzyme catalyzes the synthesis of the active folate isoform (i.e., 5-methyl-THF) efficiently involved in DNA synthesis and methylation processes [43,51]. *MTHFR* gene defects and variants have been associated with an array of complex neurological conditions [67,68].

The main investigated and clinically effective *MTHFR* single nucleotide polymorphisms (SNP) are the C677T transition (rs1801133) and the A1298C transversion (rs1801131). There is an estimate that more than 60% of the general population carries one of the two polymorphic alleles, and at least 10% of them carries both the alleles being homozygotes (677TT or 1298CC) and/or compound heterozygotes rarely in *cis* (CT/AC) more frequently in *trans* (CT/CA) in consideration of the strong linkage disequilibrium [69].

*MTHFR* C677T variant is due to a cytosine change in thymine at position 677 of exon 4 leading to the replacement of an alanine by a valine (A223V) resulting in a thermolabile enzyme with reduced enzyme activity which is particularly marked in folate deficient conditions [49,69]. There exists a strong direct correlation between folate availability and Hcy levels by distinct *MTHFR* genotypes both in normal and case patients [43,49]. For instance, the homozygous C677T (TT) condition is associated with increased Hcy and lower folate levels [31]. Accordingly, maternal folate status, mainly driven by interactions between specific genetic backgrounds and diet, is of particular relevance during pregnancy since it may favor ASD susceptibility [70,71].

*MTHFR* A1298C variant is due to an adenine change in a cytosine at position 1298 of exon 7 leading to the replacement of glutamic acid by an alanine (E429A) resulting in an enzyme with reduced activity more detrimental in the homozygous 1298CC condition though at a lesser extent than the C677T [72,73]. The association of A1298C with NTD and mental illness seems almost controversial [67]. One possible explanation for such diverse findings is that the risk for NTD might depend on the combined gene polymorphisms and/or additional genes and variants also influenced by nutritional factors [69,74].

Combined heterozygosity of the two *MTHFR* variants leads to lower *MTHFR* enzyme activity than the two single heterozygosity separately and causes high Hcy and low folate levels to an extent comparable to 677TT homozygotes [75]. *MTHFR* 677T/1298C *cis*-haplotype is a rare condition and it has been more frequently observed among spontaneous abortions than in healthy neonates suggesting strong unfavorable effects [76,77].

A previous meta-analysis reported a weak correlation between *MTHFR* C677T polymorphism, depression, and anxiety in children and adults by studying 1,119 cases of schizophrenia and 1,308 controls reporting that 677TT genotype had the greatest risk of schizophrenia (OR = 1.48; CI 95%, 1.18–1.86), compared to subjects with CC and CT genotype combined [78]. In the same study by comparing the CT genotype with CC homozygotes no significant risk association was found (OR = 1.04; CI 95%, 0.87–1.25) [78].

The finding that sex and age differently influence folate and Hcy levels and in turn cell specific methylation status ascribes to *MTHFR* genotypes a potential role on various psychiatric disorders [79]. Similarly, sex hormones show targeted effects on psychiatric disorders with a protective role of estrogen on neurodevelopment and social maturation in schizophrenia while testosterone increases male vulnerability due to its unfavorable effect on neurotransmitters regulation [79,80].

The role of *MTHFR* in ASD has been less investigated compared with other mental illnesses, such as schizophrenia and depression, though several reports highlighted a potential role of C677T and A1298C variants in ASD risk establishment (Table 1). Interestingly, *MTHFR* polymorphisms and folate status might be involved in the early phase of ASD establishment during pregnancy, as reported in both preclinical setting [80] and clinical studies (Table 1). Of interest, studies on mother-child dyads strongly support the crucial role of the crosstalk between mother/child gene landscapes and circumstantial conditions such as perinatal intake of folate supplement in different ethnicities. In particular, in the CHARGE study, a correlation between mother/child genotypes of one-carbon metabolism genes and periconceptional vitamins intake has been reported in ASD [81]. The authors highlight higher frequencies of 677TT homozygosis in ASD children than in healthy controls, and the mothers of ASD children carrying TT-genotype less likely had prenatal vitamins intake [71,81].

Although C677T transition was more frequently reported as potentially involved in ASD risk establishment, synergic effects also emerged between the two *MTHFR* SNPs (Table 1) and the reported results are encouraging, though the precise role of C677T and A1298C in ASD is still almost controversial [82]. Of note, a clinical application of genetic testing in autism has been recently attempted in a two-year-old boy at high risk of autism in which *MTHFR* genetic screening allowed an early therapeutic folate supplementation as a conventional therapeutic regimen. This approach led to a significant clinical recovery, supporting an effective pharmacogenetics approach in such a complex disease [83]. 

Overall, it becomes clear that there is the need for additional genetics and epigenetics studies preferentially focused on the mother/child dyad genome comparison also considering the low number of ASD females included in the studies.

### 2.2. DHFR Gene and Functions

The DHFR gene is located on chromosome 5 (5q14.1); it comprises 6 exons and encodes for a protein product of 187 aa [97,98]. It functionally catalyzes the conversion of DHF into THF and folic acid into DHF and THF [99]. DHF and THF are the two key folate isoforms involved in the folate cycle and Hcy metabolism as well as in de novo synthesis of a variety of essential metabolites including amino acids, lipids, pyrimidines, and purines [68]. A different DHFR enzyme activity is decisive for the relative ratio between optimal DNA methylation and faithful DNA replication [43]. One of the main functional polymorphisms within DHFR gene is a 19-base pair insertion/deletion (19bp ins/del) (rs70991108) in the promoter/first intron of the gene [68,100], associated with a limited ability of the enzyme to convert THF into 5,10-methylene THF [68]. Alongside DHFR also holds additional important non-folate-related roles in converting dihydrobiopterin (BH2) to tetrahydrobiopterin (BH4) which is the co-factor for dopamine and serotonin enzymatic production [101]. Thus, downregulation of DHFR enzymatic activity leads to decreased BH4 levels and to an imbalanced BH4/BH2 ratio that is essential for NO-synthesis, inhibition of superoxide release from endothelial NO-synthase and other functions including tyrosine hydroxylase (involved in the production of l-dopa), tryptophan hydroxylase and phenylalanine hydroxylase [101,102,103].

DHFR 19bp ins/del has been independently associated, alone or in combination with other folate related gene variants, with a significant risk for ASD, possibly by interactions between folate and the glutamatergic nervous system (Table 1) [86]. Folate isoforms conjugate indeed with glutamate residues, facilitating the excitatory properties of glutamate. For this reason, a dysregulated Hcy remethylation may affect the glutamatergic signaling since it acts as an endogenous agonist of a subgroup of excitatory glutamatergic receptors involved in the synaptic transmission. Therefore, dysregulated folate and glutamate homeostasis can be considered combined key factors in the occurrence of ASD. Accordingly, the Autism Genome Project Consortium published in the past a study on autism risk loci. The Consortium considered the glutamate-related genes as promising candidates in ASD since glutamate pathway has a key role in neuronal plasticity and development suggesting that ASD could be considered a glutamatergic system disorder [104].

Of note, the use of DHFR inhibitors in oncologic children points out great concerns due to potential long-term side effects. In detail, the folate antagonist methotrexate, by lowering THF availability and in turn pyrimidine and purines production as well as RNA and DNA synthesis, has been associated with decreased volume of subcortical structures, cognitive impairment and increased prevalence of autistic-like symptoms among methotrexate treated children [105,106].

It has been also reported that folic acid supplements might saturate DHFR enzyme in the liver of humans, and then slow down the conversion of folic acid to THF taking up to 12 h for a single 5mg dose of folic acid in selected individuals carrying susceptible haplotypes, suggesting caution regarding over-supplementation [107,108]. Concern has been expressed particularly about unmetabolized folic acid (UMFA) that may be detrimental in the presence of specific haplotypes, particularly in pregnant women carrying selected folate gene variants [109]. In fact, during pregnancy, unbalanced folate isoforms distribution may drive and favor aberrant epigenetic mechanisms on the offspring, and nonetheless, maternal folate supplementation efficiently reduces congenital malformations as NTD or cleft palate, indiscriminate fortification may cause severe pediatric pathological conditions [109,110]. A suboptimal conversion of folic acid into active folate, as in presence of particular *DHFR* and *MTHFR* gene variants, reduces UMFA-threshold particularly during critical conditions such as pregnancy, exacerbating negative side effects and concerns, suggesting a a safe supplementation assessed by pharmacogenetics investigations [108,111].

In the context of ASD, DHFR 19bp ins/del has been suggested as an inherited modifying factor during pregnancy, mainly due to an unusual DHFR activity associated with unmetabolized pteroylmonoglutamate (i.e., therapeutic folate) during the embryogenesis process by accumulation in the central nervous system [109]. Accordingly, a positive correlation has been found between *MTHFR* C677T and *DHFR* 19bp ins/del in ASD individuals [86] evoking changes in the brain epigenome. The authors concluded that although folate status and/or associated genes might not be the direct cause of ASD, environment (i.e., nutrients)-gene interactions by affecting other gene products might modify ASD risk mainly due to a comprehensive effect of folate machinery on the whole genome [86].

Finally, a recent multicentric study is aiming at determining if reducing folic acid supplementation during late gestation might also reduce maternal UMFA, considered a risk for ASD as well childhood allergy and metabolic diseases [112]. Other studies investigated the potential association between high folate levels in maternal blood and ASD focusing on whether different kinds of folate in cord blood could have specific associations with ASD, reporting that higher UMFA concentrations in cord blood but not 5-methyl-THF or total folate were associated with increased risk of ASD in black children [31,96].

Overall, though considering the potential risk associated with specific *DHFR* and *MTHFR* haplotypes, and in the light of the recent associations found by the research group in the Genetic-Epigenetic-Mother-Child-Dyad-Study (GEMCDS) that discovered unexpected opposite effects on the onset age of pediatric leukemia according to specific haplotypes carried by the mother or the child [51], further investigations are strongly warranted. Then, before assigning or refusing a definite association, often controversial in the literature, both mother and child dyad genomes must be taken into account [96] to properly readdress a targeted periconceptional use of supplementation.

### 2.3. CBS Gene and Functions

*CBS* gene is located on chromosome 21 (21q22.3), it comprises a total of 23 exons and codes for a protein product of 551 aa [113]. The gene contains alternative exons 1 (exons la-le) and other exons defined by multiple alternatively spliced transcripts encoding the *CBS* protein [113]. The 5′UTR contains one of five alternatively used exons and one constantly present exon, while 3′UTR is encoded by exons 16 and 17 [113]. The protein is organized as a homotetramer of 63 kDa subunits and each subunit binds two substrates (i.e., homocysteine and serine) [114] to catalyze the irreversible metabolization of Hcy to cysteine. It requires vitamin B6 as an essential cofactor [115] and by means of the transsulfuration pathway [116,117] irreversibly removes Hcy from the methionine cycle lowering, in turn, Hcy from circulation [117]. Different from Hcy, cysteine can be taken from the diet, if cysteine supply is high, the oxidative/desulfuration pathways may result unbalanced. Briefly, high Hcy causes redox imbalance and oxidative stress with free radical release, while cysteine being an antioxidant strongly contrasts oxidative damage also affecting DNA, lipids, and proteins highlighting a role of the ASD/imprinting/epigenetic/disorders axis [118,119].

Of note, cysteine is fundamental for protein production and for glutathione generation, the potent mediator with antioxidant and detoxifying effects against xenobiotics [115]. Very high circulating Hcy levels are risk factors for different pathological situations and levels above 50–100 μmol/L are considered an intermediate-severe condition [59]. An intra-individual variability, due to the presence of the main gene variants described above, is often described and individual genetics is globally involved in the final Hcy blood concentration.

Functional defects of the *CBS* enzyme cause classical homocystinuria, and associations have been demonstrated between altered methionine/homocysteine metabolism and cognitive or behavioral diseases, ASD included [120]. *CBS* gene defects are associated with reduction of normal vascular functions, increased systemic oxidative stress, brain atrophy, and worsening of the neurological impairment in various central nervous system disorders, *in primis* autism, epilepsy, Parkinson’s disease, Alzheimer’s disease, and dementia [101,120,121]. In a study on ASD children aimed at identifying biomarkers of increased oxidative stress and impaired methylation score, lower blood levels of methionine, SAM, Hcy, cystathionine, cysteine, and total glutathione and higher blood levels of SAH, adenosine, and oxidized glutathione have been found potentially associated with behavioral disturbances [120]. Authors observed that anomalies in the metabolic profile, particularly in the transsulfuration pathway, could be responsible for higher susceptibility to environmental and/or to cellular oxidative stress and impaired methylation capability, responsible for the clinical phenotype [120]. As stated above, a decreased *CBS* activity, controlled by methionine and SAM, will increase the cysteine requirement, leading to a decrease in total glutathione concentrations, suggesting that ASD patients are more susceptible and less protected against oxidative stress, also in an epigenetic perspective [118,119,120]. Uncontrolled and unbalanced oxidative stress, also exacerbated by increased local tissue iron deposits, is a crucial factor in determining tissue damage and cognitive decline, and as recently found it is strictly directed by genetic interactions of iron homeostasis genes and *APOE* haplotypes [23,122,123].

More than one-hundred different mutations, prevalently clustered in exon 3, 8, and 10, have been reported in the *CBS* gene as causative of complex diseases [101,113]. In a study on schizophrenia, it has been reported an association between the common *CBS* polymorphism of 68-bp insertion (844ins68) with increased disease risk [124]. Other studies demonstrated an association of *CBS* polymorphisms with ASD children [93] as the C699T variant (rs234706) in which 699TT-homozygotes and CT-heterozygotes were significantly more represented among ASD cases compared to healthy controls with the polymorphism playing also a role in sleep and gastrointestinal disorders [93]. Interestingly, the frequency of the T-allele had a significant association with the high score of the Childhood Autism Rating Scale (CARS) and with other clinical data related to ASD participants [93].

Finally, a recent study on premature infants with encephalopathy occurring in the setting of hypoxia-ischemia, suggested that neonatal brain injury and long-term damage was due to *CBS* upregulation, highlighting this pathway as a potential molecular target to counteract encephalopathy in premature infants also taking into account that up to 50% of these children showed ASD [125].

## 3. One-Carbon Metabolism in Autism and in Other Neurological Diseases: Brain Sex-Related Insights

In many complex diseases, neurological conditions included, sex differences are associated with prevalence, course of the disease and outcome [126,127]. Among behavioral abnormalities, epidemiological studies consistently reported higher ASD prevalence in males compared with females (male: female ratio about 4:1), also considering the different role of abnormalities detected in the mother or father of ASD children [16,126,128,129]. Moreover, ASD females show a less severe disease phenotype highlighting sex differences also in a genetic view [130].

GWAS are demonstrating that including balanced sexes in the recruitment and analyses and stratifying data by sex strongly improved the comprehension and the ability to transfer experimental data in the clinical practice [131]. In a wide prospective study of newly diagnosed preschool ASD children, it was observed that the sex of the affected child with ASD was the only significant predictor of differential trajectories of symptoms over time [132]. Boys had more stable, severe symptoms over time, whereas girls exhibited less severe symptoms and improvement over time [132] in accordance with the evidence that some girls no longer showed cognitive and language problems at follow-up [132]. 

An increased sex gap was also present when considering prevalence among very young patients [16]. The male-to-female ratio was 1.16:1.0 when using an at-risk sample of infants ranging from 22 to 39 months [133,134]. Moreover, research also highlighted that the reported ratio was affected by the level of cognitive ability and the greater the attention defects the less the difference between male/female ratios was apparent [133]. Finally, infants aged 70–75 months had an overall male-to-female ratio of 2.61:1.0, and again when testing only those with an IQ below 50 the male-to-female ratio fell to 1.31:1.0 [133,134]. Therefore, it would also be important to examine symptom differences between sexes, also considering the specific developmental level [135]. 

Furthermore, autistic male and female patients have divergent peculiar symptoms with males displaying heightened aggressiveness and repetitive behavior, while females experiencing greater anxiety and depression [136]. There is also a discrepancy when diagnosing a male child with autism compared to a female child, due to the differences in the symptom picture, which still has in part a male-centered component in the official criteria to diagnose and globally this contributes to a preferentially earlier diagnosis in male children [137,138].

Since there exists a clear difference in autism between sexes, this points out to differences in the biological pathways underlying ASD development in the two sexes [134,136]. From a molecular perspective, defects in folate metabolism can be targeted to achieve a sex specific prevention program, as well as treatment and therapy [12]. As a paradigm of this approach, in a different clinical context it has been shown that *MTHFR* C677T polymorphism had different methylation effects stratified by sex in patients with schizophrenia with female patients showing a tendency towards lower rates of global methylation [139]. The combination of sex and other variables correlated with global methylation revealed that sex and *MTHFR* genotype strongly interacted, ascribing to 677 TT-homozygous females the lowest overall methylation rates compared to males [139]. 

Interestingly, preclinical sex-oriented researches demonstrated correlations between *MTFHR* polymorphic status and observed behaviors in mice [140]. In particular, newborn mice with *MTHFR* 677TT genotype when exposed to antiepileptic drugs were positively associated with higher altered social behaviors differently expressed among female and male mice [140]. This behavioral outcome was correlated with different cortical potency of reeling level, and with altered proportions of key proteins involved in the excitation/inhibition synapses in the brain of female mice [140].

**Figure 2:**
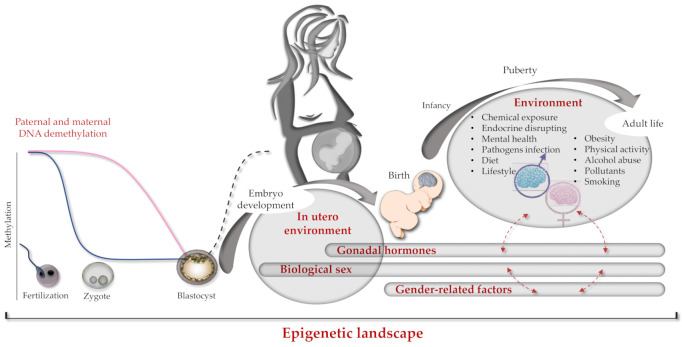
Brain epigenome imprinting. Schematic representation of genetics and epigenetics interactions occurring from conception to individual adult life. On the left, paternal and maternal hemigenome DNA demethylation processes occurring during fertilization (blue and pink lines respectively). In the middle, de novo global genome methylation at the blastocyst phase is shown as a dark dashed line. On the right, dashed red arrows indicate cross-interactions between different epigenetics factors (e.g., sex and gender).

Information and experimental data on sex differences between human brain developments according to the brain epigenome are very limited. Since some of such differences are evident in the brain before birth, they should happen during pregnancy in which sex imprinting actions can be mainly ascribed to the mother and less to the developing fetus. After birth, the genome, epigenome, and gender of the newborn take place, mutually interacting in determining the ultimate brain epigenome (Figure 2).

## 4. The Role of Epigenetics and Genetics: The Paradigm of the Folate Cycle

Epigenetics may provide a different view to the mechanisms and insights of the pathophysiological processes in complex diseases such as developmental diseases, potentially leading to the identification of innovative therapeutic targets and strategies [141]. In a few words, epigenetics can be resumed as modifications of heritable phenotype variations with no alterations of the DNA sequence, and epigenome dysregulations have been recognized as hallmarks in several diseases. In mammalians, epigenetic modifications influence transgenerational inheritance by DNA methylation and histone modifications (i.e., methylation, acetylation, phosphorylation, sumoylation, and histone gene variants) together with small and long non-coding RNAs (i.e., micro-RNA) [19,142]. Epigenetics emerges as a dynamic and reversible process occurring in multiple rounds with a key role of modifications during the first phases of embryonic development when parental genetic/epigenetic marks can be inherited by the offspring as recently reviewed [142]. Strong sex differences already start at the zygote stage by the completely different methylation process occurring within the mother and father hemigenomes: at an early zygote status for the paternal hemigenome, characterized by an active enzymatic dependent methylation erasure before DNA replication, and by a slower rate of spontaneous methylation erasure for the maternal hemigenome during the following cell divisions (Figure 2). This complex process is measurable by assessing the relative ratio of 5-methyl-cytosine and 5-hydroxy-methyl-cytosine (5mC/5hmC) the latter being the first oxidative product in the active demethylation of 5mC [143,144].

Basically, in the brain, a de novo DNA methylation driven by DNA methyltransferases (DNMTs) is necessary for learning and memory activities and the methylation degree is linked to specific neural activity [145]. Interestingly, brain DNA contains 5hmC and whole genome bisulfite sequencing (WGBS) has shown elevated percentages of non-CpG methylated residues revealing that the most common base substitution is an adenine (i.e., mCA). Global methylome reorganization occurs during fetal/early-childhood development, and during this period highly conserved non-CpG methylation (i.e., mCH) becomes the prevalent form of methylation in the human brain genome [146]. This modification prevails during postnatal development of the brain concomitantly with synaptogenesis and circumstantial personal experiences altogether strongly contributing to define cell identity with possible alterations during perinatal estradiol exposition.

Since epigenetic modifications control how and to what extent genes must be expressed, and respond to the environment stimuli, any disturbance of the normal interplay between environmental factors and epigenetic reprogramming may result in the occurrence of specific disease conditions. This mechanism has been suggested for autism initiation/progression including the onset-age. In a recent study, 84 rare epigenetic variations (epivariations) have been identified in autism compared to healthy controls [147], with a trend of epivariations to cluster in affected autistic patients compared to unaffected brothers/sisters within the autism families [147]. Many genetic and epigenetic factors potentially involved in ASD, as well as the crosstalk between major gut microbiota metabolites in autistic children and epigenetic changes, have also been recently discussed and reviewed [148,149]. Interestingly, by investigating the different aberrant DNA methylation degrees, an important sex-related difference between autistic children and sex-matched non-autistic siblings has been found [150]. In particular, the authors reported that some sex-specific methylation patterns (linked to mitochondrial dysfunction and metabolic disorders) may provide a degree of protection against autism in females, highlighting sex-specific epigenetic traits that require dedicated investigations [150]. Of note, sex-based epigenetic differences (i.e., methylation rate) on key genes, such as the oxytocin receptor gene *OXTR*, have also been recently reported [151]. However, the mechanisms underlying these epigenetic changes are still unclear and strongly heterogeneous. The suggested critical processes are related to changes in levels of transcription of key genes during specific crucial phases of central nervous system development, also affected by genetic and/or environmental dynamics.

Since it has been demonstrated that several environmental factors including progenitor’s diet and lifestyle can influence the inherited epigenetics trait [142,152,153], the folate and folate cycle may represent a paradigmatic example of the inception of epigenetics in response to environmental variances. As mentioned above, crucial epigenetic reprogramming occurs during the first stages of embryo development, starting from the fertilization step with potential effects till the stage of morula/blastocyst when the reconstituted diploid genome begins the remethylation program [154]. Accordingly, maternal/paternal folate status due to both genetically inherited predispositions and/or folate intake might differentially affect the global embryonic DNA methylation program [51,155]. Of note, in a preclinical model of autism it has been recently demonstrated that epigenetic dysregulation (i.e., hypomethylation) of key genes (brain-derived neurotrophic factor gene *BDNF* and glial fibrillary acidic protein *GFAP* gene) involved in the induced autistic-like behaviors in a neonatal isolation model, can be reversed by folic acid administration [156]. Interestingly, the treatment effects were mediated by the epigenetic regulation of *BDNF* and *GFAP* by restoring the hypomethylated status of the two gene promoters and by antioxidant effects, opening stimulating translational applications.

Histone methylation processes by histone methyltransferases (HMTs) are a crucial part of the maternal influence effects on offspring. This may result via the epigenetic code regulation of chromatin status (including chromatic reorganization), affecting in turn gene expression [152]. HMT activities depend on intracellular SAM levels and HMT gene variants, connecting mother-child metabolism and cell nutrient availability [19,157]. Folate deficiency and one-carbon metabolism genes also result in altered epigenetic histone modifications and during pregnancy, they have been correlated with increased risk of neurocognitive and/or neurobehavioral deficits such as ASD and attention deficit hyperactivity disorders [158]. Finally, modified histones are secreted in the uterine environment and may influence embryo brain development by transgenerational epigenetic histone modifications [159].

## 5. Conclusions and Future Perspectives in ASD

**Figure 3:**
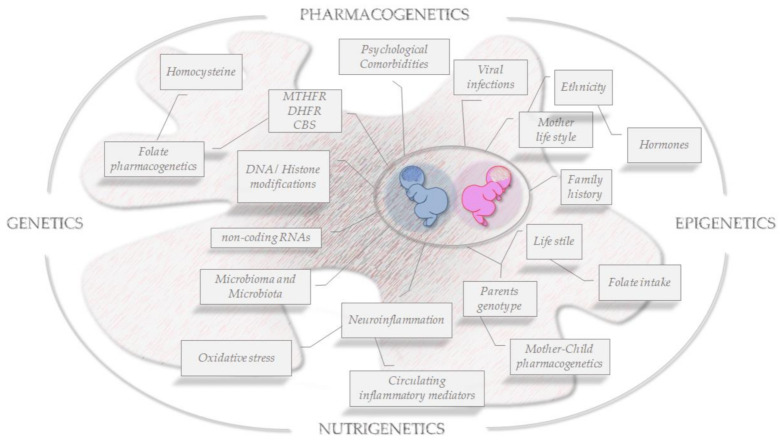
Folate OMICS-environment interactions on brain epigenomics. Snapshot of key risk factors for ASD development and key transgenerational pathophysiological features to be faced in a sex/gender approach.

Although the key role of folate isoforms balance/dysregulation in some complex pathological conditions has been demonstrated, more efforts and dedicated studies are needed to conclusively identify the most critical factors involved in ASD establishment. In particular, there is the mandatory need to elucidate the precise mechanisms by which sex-specific factors can modulate disease onset, severity and the different disease phenotypes at presentation, as well as how they can help and guide the choice of useful or unsafe dedicated treatments. To face these challenges, the synergy between advanced technologies and experimental/clinical investigations including pharmacogenetics/genomics/OMICS approaches [160,161,162,163] and personalized medicine [164] will allow a progression in the pathophysiological understanding of complex neurodevelopmental/neurological diseases. Earlier identification of informative molecular and biological biomarkers and an appropriate therapeutic strategy definition are the unique effective strategy to follow (Figure 3). Accordingly, the sex gap in ASD prevalence reported to be about 4:1 (males-to-females respectively) appears to be more realistically close to 3:1 due to a potential diagnostic gender bias that does not efficiently include ASD girls, that are therefore at high risk of not receiving a proper and early diagnosis [165].

In conclusion, due to an unavoidable mutual influence between the psychological/social gender and the biological sex in human life, and that they both interact on the brain development by genetics and epigenetics actions, we cannot easily separate the effect of sex or gender on the brain epigenome establishment. Then we must begin to consider sex and gender combined together any time a difference appears in the clinical phenotype between women and men [166].

## Figures and Tables

**Table 1:** Selected Studies on *MTHFR*, *DHFR* and *CBS* Genes Reporting Significant Associations with ASD.

Gene	Key Findings	Genotype/Allele	Ref
*MTHFR*	• Increased frequency of *MTHFR* C677T in ASD • Unexpected high frequency of the normal *MTHFR* 1298AA genotype in ASD • Combined *MTHFR* 677CT/1298AC haplotype more prevalent in ASD	677 CT/TT 1298 AA	[84]
*MTHFR*	• Circulating methionine and SAM/SAH ratio are significantly decreased in ASD • Circulating cysteine, GSH, and GSH/GSSG ratio are significantly decreased in ASD • Disease association with *MTHFR* C677T and A1298C	677 CT+TT 1298/677 AC/CT combined with *RFC* 80GA	[85]
*DHFR*	• *DHFR* 19bp ins/del is a risk factor for ASD independently from and in association with folate polymorphisms • *DHFR* 19bp ins/del combined with *MTHFR* C677T and A1298C	19bp del 19bp del+677T+1298C	[86]
*MTHFR*	• *MTHFR* C677T emerges as primary ASD risk factor • *MTHFR* A1298C emerges as additive risk factor for ASD in combination to C677T	677 T 677 T + 1298C	[72]
*MTHFR*	• High frequency of *MTHFR* 677 T-allele and TT-genotype, 677/1298 T/A and TT/AA haplotypes in ASD • Preferential parental transmission of 677 T- and 1298 A-allele or 677/1298 T/A haplotypes in affected offspring	677 T 1298 AA	[87]
*MTHFR* *CBS*	• Periconceptional vitamins intake reduces the risk of having ASD children in genetically susceptible mothers/children dyad • Higher ASD risk in mother *MTHFR* 677TT, *CBS* rs234715 GT+TT with child *COMT* 472 AA genotypes • Higher ASD risk in mothers also carrying other one-carbon metabolism gene variants	677 TT combined with other one-carbon gene variants, both in mother and child	[81]
*MTHFR*	• Lower ASD risk associated to folic acid supplement strongest in *MTHFR* C677T carriers (mothers/children)	677 CT+TT	[88]
*MTHFR*	• High frequency of *MTHFR* 677TT in ASD children • Over-activity significantly associated to *MTHFR* 677TT genotype (Stratification by Autism Diagnostic Interview)	677 TT	[89]
*MTHFR*	• Meta-analysis: eight case-control studies included • Higher ASD risk to *MTHFR* C677T polymorphism (all comparison models) • Lower ASD risk to *MTHFR* A1298C polymorphism (recessive model) • ASD association to *MTHFR* C677T polymorphism (only in countries without food fortification)	677 CT+TT 1298 CC	[71]
*MTHFR*	• Associations to ASD with *MTHFR* A1298C • Higher ASD risk to *MTHFR* 677CT/1298AC combined genotype • No significant associations in females	1298 AC+CC 677/1298 CT/AC	[90]
*MTHFR*	• Meta-analysis: thirteen studies included (9 on Caucasians, 4 on Asians) • Significant association between ASD and *MTHFR* C677T polymorphism	677 CT+TT 677 TT 677 T	[91]
*MTHFR*	• Higher *MTHFR* A1298C frequency in ASD (AC: 41.9%; CC: 35.5%) • Higher *MTHFR* C677T frequency in ASD (CT: 48.4%; TT 12.9%) • Heterozygosity was equally detected (46.2%) among patients with severe autism	677 CT+TT 677 T 1298 AC+CC 1298 C	[92]
*CBS*	• Higher *CBS* C699T frequencies distributions (TT and CT+TT) in ASD patients • Lower *CBS* C699T frequency associated with sleep and GIT disorders • No significant association between *CBS* genotypes and severity of ASD	699 CT+TT 699 TT	[93]
*MTHFR*	• Meta-analysis: 25 case-control studies on *MTHFR* (C677T, 18 studies) (A1298C, 7 studies) • Higher *MTHFR* C677T frequency in ASD • No overall association between *MTHFR* A1298C and ASD risk MTHFR A1298C significantly associated only in Caucasians	677 CT+TT 677 TT 677 T 1298 CC 1298 C	[94]
*MTHFR*	• Meta-analysis: 15 studies • Higher ASD risk to *MTHFR* C677T polymorphism (all comparison models) • No association between *MTHFR* A1298C and ASD (all comparison models)	677 CT+TT 677 TT 677 T 677/1298 T/C	[95]
*DHFR*	• Positive association (not adjusted) between cord total folate and UMFA also after *DHFR* genotype stratification (limited to Black children)	19bp del/del	[96]

## Data Availability

Data is contained within the article.
